# Associations of clustered health risk behaviors with diabetes and hypertension in White, Black, Hispanic, and Asian American adults

**DOI:** 10.1186/s12889-022-12938-y

**Published:** 2022-04-15

**Authors:** Won Kim Cook, Libo Li, Christina C. Tam, Nina Mulia, William C. Kerr

**Affiliations:** Alcohol Research Group, Public Health Institute, 6001 Shellmound St. Suite 450, CA 94608 Emeryville, USA

**Keywords:** Health behavior, Life style, Diabetes mellitus, Hypertension

## Abstract

**Background:**

The clustering of Big Four contributors to morbidity and mortality—alcohol misuse, smoking, poor diet, and physical inactivity—may further elevate chronic health risk, but there is limited information about their specific combinations and associated health risks for racial/ethnic minority groups. We aimed to examine patterns of clustering in risk behaviors for White, Black, Hispanic, and Asian American adults and their associations with diabetes and hypertension. As these behaviors may be socioeconomically-patterned, we also examined associations between clustering and socioeconomic status (SES).

**Methods:**

Latent class analyses and multinomial and logistic regressions were conducted using a nationally-representative sample of United States (US) adults ages 40–70 (*N* = 35,322) from Waves 2 (2004–2005) and 3 (2012–2013) of the National Epidemiologic Survey on Alcohol and Related Conditions. Obesity was used as a proxy for unhealthy diet. The outcomes were diabetes and hypertension.

**Results:**

A *relatively-healthy-lifestyle* class was found only among White adults. Common patterns of unhealthy clustering were found across groups with some variations: the *obese-inactive* class among White, Black, and Hispanic adults (and the *inactive* class among Asian adults); the *obese-inactive-smoking* class among White, Black, and Hispanic adults; the *smoking-risky-drinking* class among White and Hispanic adults; and the *smoking-risky-drinking-inactive* class among Black and Asian adults. Positive associations of unhealthier clustering (having a greater number of risk behaviors) with lower SES (i.e., family income and education) and with health conditions were more consistent for Whites than for other groups. For racial minority groups, lower education than income was more consistently associated with unhealthy clusters. The associations between unhealthier clustering and diabetes and hypertension were less clear for Blacks and Asians than for Whites, with no significant association observed for Hispanics.

**Conclusion:**

Concerted efforts to address clustered risk behaviors in most US adults, particularly in racial/ethnic minority groups given the high prevalence of unhealthy clustering, are warranted.

## Introduction

There has been increasing research interest in lifestyle risk behaviors that collectively increase health risk. Of particular concern are alcohol misuse, cigarette smoking, poor diet, and physical inactivity—the “Big Four” contributors to mortality [1] and the leading proximal and modifiable causes of morbidity [2]—in which their synergistic effects are suggested to be more detrimental to health than their cumulative individual effects [2]. Still, there is a paucity of information about the specific combinations of them [3], as studies have focused on quantifying the co-occurrence of these behaviors, mostly using counts of risk behaviors [4] or lifestyle indices as summary measures of healthfulness of lifestyles [5, 6].

In the current study, we aim to examine the clustering of these behaviors and its associations with two common chronic conditions, hypertension and diabetes, among White, Black, Hispanic, and Asian American adults. Hypertension is the leading single risk factor for morbidity and mortality, and a major risk factor for cardiovascular disease [7, 8], the leading cause of death in the United States (US) [9]. Type 2 diabetes is accompanied by complications like cardiovascular diseases, retinopathy, nephropathy, and cancers, and consequently associated with increased risk for premature death [5]. Although genetic predisposition partly determines individual susceptibility, these conditions largely are by-products of unhealthy lifestyles featuring health risk behaviors [10]. Continued engagement in these behaviors while having these conditions increases the risks for complications with greater morbidity and premature mortality.

Of note, race-specific information about the clustering of these behaviors is rare. To our knowledge, our recent study is the first one that has reported on the clustering of the Big Four behaviors among Whites, Blacks, and Hispanics, using the National Alcohol Survey data and validating the clusters using self-rated health as the outcome [11]. Health behaviors are influenced by the sociocultural and economic circumstances that shape decisions about them [12, 13]. Racial/ethnic minorities (excepting Asians) tend to have lower SES than Whites in the US [14]. As past research suggests that individuals of lower SES are more likely to engage in unhealthy behaviors such as tobacco use, physical inactivity, and poor nutrition [15], more unhealthy clustering of lifestyle behaviors may be observed among Black and Hispanic adults.

There is also evidence that cultural norms and expectations in ethnic minority communities influence health behaviors. For example, in addition to income, prices, and access to quality fresh food (often dictated by one’s SES), dietary patterns are also shaped by individual preferences and beliefs, and sociocultural and ethnic factors [16, 17], with social norms and modeling exerting powerful influences on food choice and consumption amounts [18–21]. Foods are often used to affirm culture and forge social bonds, and frequent kinship gathering among Blacks and Hispanics around food, where rich, traditional or cultural foods may take precedence over more healthful eating, along with community norms that may dissuade adopting healthier food options [17, 22], may also result in unhealthier diets being more pervasive in Black and Hispanic communities. Low social support for health-promoting activities in Black communities such as healthy diet or regular exercise has also been noted as a barrier to a healthy lifestyle [23, 24], and might reflect a lack of neighborhood amenities (e.g., recreational spaces, affordable and accessible fresh foods) fostering this [25, 26]. Asian cultural values that do not prioritize physical activity [27] may contribute to a sedentary lifestyle. Additionally, greater exposure to stressors associated with racial minority status such as racial discrimination [28] and lower access to health-promoting resources such as health care may lead to unhealthier lifestyles among racial minority groups. The clustering of health risk behaviors, therefore, is likely to be ethnically-patterned, and understanding race-specific patterns is critical for informing contextually-relevant interventions.

As these risk behaviors constitute pathways that lead to disparities in these conditions or their management [29], a better understanding of their clustering, common or varying among these groups, can inform appropriate intervention strategies tailored to each group. As the effects of race and SES on health are confounded in the US [30], to disentangle the respective effects of race and SES, each of which may engender constraints on health-related behaviors, we also examine whether unhealthier clustering is associated with lower SES in each group.

Three research questions are addressed: 1) What are the common and diverging patterns of clustered risk behaviors across these four racial/ethnic groups?; 2) Is unhealthier clustering associated with lower SES?; and 3) Is unhealthier clustering associated with diabetes and hypertension?

## Methods

### Data

A nationally-representative sample of US adults ages 40 to 70 (*N*= 35,322) was drawn from Waves 2 (2004–2005) and 3 (2012–2013) of the cross-sectional National Epidemiologic Survey on Alcohol and Related Conditions (NESARC). This sample excludes younger and older age groups because of age-related health risks and lifestyle patterns. Diabetes and hypertension become more prevalent in midlife, with their risks steeply increasing in older adulthood largely due to aging [31]. Older adults tend to reduce dietary/alcohol intake, quit smoking out of health concerns, and reduce physical activities due to age-related functional declines [32–34]. NESARC used multistage probability sampling, and the response rates were 87.6% at Wave 2 and 84.0% for Wave 3.

### Measures

#### Lifestyle factors

In light of research showing beneficial effects of moderate drinking on some chronic conditions (e.g., diabetes) and adverse health effects of heavy drinking [35–37] and abstinence [36, 38], past-year alcohol consumption was an ordinal variable of lifetime abstinence, former drinking, drinking < 7 drinks/week, 7- < 14 drinks/week, and > 14 drinks/week (hereafter referred to as *risky drinking*), based on the low-risk drinking guidelines of the National Institute on Alcohol Abuse and Alcoholism [39].

*Smoking status* had three categories of current smoker, former smoker, and lifetime non-smoker [40–42] to differentiate former smoker who may have quit smoking due to health concerns from lifetime non-smoker.

With no information about diet in NESARC, *obesity* (a body mass index of > 30 kg/m^2^) [43] was used. Obesity is an indicator of a state of positive energy balance that reflects chronic overeating [44] and attributed primarily to excess caloric intake [45], and thus is a reasonable proxy for unhealthy diet. Importantly, obesity is among the most prominent risk factors for a host of debilitating and life-threatening chronic conditions such as type 2 diabetes, cardiovascular diseases, and some cancers [46].

*Physical inactivity* is a dichotomous variable of < 150 min of moderate-intensity or < 75 min of vigorous-intensity activity weekly, based upon the US guidelines for physical activity [47].

#### Health outcomes

A dichotomous measure of doctor-diagnosed and self-reported *diabetes* or *hypertension* was based upon affirmative responses to both of the two questions: “During the last 12 months, did you have [name of condition]?”; if yes, “Did a doctor or other health professional tell you that you had [name of condition]?”

#### Demographic variables

Race/ethnicity was assessed using two items: one for selecting 1+ categories that describe the respondent’s race among Whites, Blacks, and Asians; and another about the respondent’s being of Hispanic or Latino origin.

*Marital status* variable indicated being married/living with a partner versus widowed/divorced/separated/never married [11]. Marital status was associated with both health outcomes [48] and health behaviors [49, 50].

*Education* was a dichotomous variable of having a 4-year college or advanced degree versus less than a college degree. *Family income* was a ratio of family income to the corresponding survey year’s US Federal Poverty Level [51]. We used separate indicators of SES to avoid the conceptual blurring of explanatory mechanisms for SES effects that occurs with use of a composite [52]. *Health insurance coverage*, which may influence disease diagnosis [53], indicated having coverage *(*versus no coverage) in the past year.

*Nativity status* (US-born versus foreign-born) and *ethnicity* based on the respondents’ countries of origin, both of which are potential confounders of the relationship between disease conditions and health risk behaviors, were included in models for Asians (using the categories of Chinese, Filipino, Japanese, Korean, South Asian, Vietnamese, Southeast Asian other than Vietnamese, and other Asian) and Hispanics (Cuban, Mexican, Puerto Rican, South American and Central American). Though research is somewhat mixed [54], cardiovascular risk factors including diabetes and hypertension were associated with US-born status for Hispanic adults [55] and Asian adults [56, 57] living in the US, as well as their ethnicity (or national origin) [54, 58]. Being US-born was associated with health risk behaviors such as obesity [56, 57] and alcohol consumption [59, 60] in these populations, as was their national origin [60–62].

### Statistical analyses

Latent class analysis (LCA), a semi-parametric statistical technique that groups individuals into mutually-exclusive and substantively-meaningful latent classes [63–65], was conducted in Mplus [66] to identify clusters of risk behaviors. Mplus is a statistical software package that can implement a wide array of statistical models, but it is primarily known for its latent variable modeling capabilities [67].

As qualitatively distinct patterns of clustering were anticipated across racial/ethnic groups, LCA was performed separately for each group to compare patterns of clustering qualitatively, not to compare latent class prevalence directly across groups [64]. Model selection was based on model fit indices and statistics (Bayesian information criterion (BIC), Akaike information criterion (AIC), sample-sized adjusted BIC (aBIC), and bootstrapped likelihood ratio tests) and practical usefulness of each class (> 5% of the sample and meaningfully differentiating an additional class) [65, 68, 69]. Our fit statistics and practical criteria, taken together, suggest a 4-class model as the most parsimonious and substantively sound for Whites and a 3-class model each for Asians, Blacks, and Hispanics (Table 2). Where AIC, BIC, and aBIC pointed to different models, BIC and aBIC were prioritized in model selection [65, 70]. We then performed logistic regressions to estimate associations between class membership and health conditions, and multinomial logistic regressions to examine the associations of class membership with SES, accounting for demographic variables. We used the 3-step method [71], which is considered superior to the standard approach of combining the latent class model and the latent regression model into a joint model [71], specifying class membership based on the maximum posterior probabilities from the best-fitting model as a nominal variable and then using the logit of this variable to estimate logistic regression models. To adjust for sampling strategy and nonresponse, sampling weights were incorporated in our model estimation.

## Results

Table 1 shows the characteristics of our sample. White adults were slightly older than other groups. Whites had the highest average income (on average 446.9% above the Federal Poverty Line), and Asians had the highest education level, with almost half (48.5%) having a 4-year college degree. Prevalence of individuals who drank more than 14 drinks per week was higher among Blacks (8.1%) than other groups. Current smoking was more prevalent among Whites (27.5%) and Blacks (27.3%) than the other two groups, and obesity more prevalent among Blacks (44.7%) and Hispanics (37.5%) than others. Asians were less likely to engage in other risk behaviors but more likely to be inactive (39.4%) than other groups. The proportion of individuals with diagnosed diabetes or hypertension, particularly the latter, was higher among Blacks (17.1% for diabetes and 25.9% for hypertension) than among other groups.

**Table 1:** Sociodemographic characteristics of US adults ages 40–70, NESARC Waves 2 and 3

	All (% (n))*	Whites (%)	Blacks (%)	Hispanics (%)	Asians (%)	*p*
Male	44.0 (15,480)	49.0	45.0	48.3	46.4	**
Age (M/SD)	53.3 (0.07)	53.8 (7.7)	52.5 (11.3)	51.2 (10.2)	51.9 (7.2)	***
College degree+	30.8 (9759)	33.6	19.5	15.9	48.5	***
% above Poverty (M/SD)	403.6 (4.5)	446.9 (291.9)	281.4 (336.4)	260.0 (291.7)	397.2 (277.8)	***
Married	69.6 (19,713)	72.3	47.7	68.4	81.9	***
Insured	82.6 (29,050)	84.8	80.2	67.4	81.0	***
Alcohol consumption						
Lifetime abstainer	10.3 (3991)	7.3	15.8	16.0	29.7	***
Former drinker	21.0 (7831)	20.2	26.8	22.2	17.5	
< 7 drinks/week	54.9 (18,502)	57.8	43.6	51.1	46.6	
7- < 14 drinks/week	6.9 (2355)	7.5	5.8	5.2	3.0	
> 14 drinks/week	7.0 (2498)	7.2	8.1	5.7	3.1	
Current Smoker	25.7 (9171)	27.5	27.3	17.3	13.8	***
Former Smoker	25.2 (8349)	28.1	17.9	19.7	12.5	
Obese	33.7 (12,113)	32.9	44.7	37.5	10.9	***
Inactive	32.3 (12,067)	30.5	38.2	34.9	39.4	***
Diabetes	4284 (12.1)	9.8	17.1	14.7	10.4	***
Hypertension	11,327 (32.2)	29.6	45.4	25.9	26.3	***
US Nativity	85.3 (29,333)	95.8	90.0	39.8	18.2	***
Hispanic ethnicity^a^						
Mexican		–	–	63.4	–	
Puerto Rican		–	–	14.7	–	
Cuban		–	–	6.1	–	
Central American		–	–	5.5	–	
South American		–	–	10.3	–	
Asian ethnicity^b^						
Chinese		–	–	–	24.8	
Filipino		–	–	–	20.4	
South Asian		–	–	–	20.0	
Japanese		–	–	–	9.2	
Korean		–	–	–	8.0	
Vietnamese		–	–	–	10.2	
Southeast Asian		–	–	–	5.8	
Other		–	–	–	1.6	

^a^Central American ethnicities include Nicaraguan, Guatemalan, Belizean, Costa Rican, and Panamanian

South American ethnicities include Brazilian, Chilean, Columbian

^b^South Asian ethnicities include Indian, Afghanistani, and Pakistani

Southeast Asian includes Cambodian, Laotian, and Thai

^*^Weighted percentages and unweighted n’s

^***^p < .001

^**^p < .01

### Clustering of health risk behaviors: latent class models

**Fig. 1:**
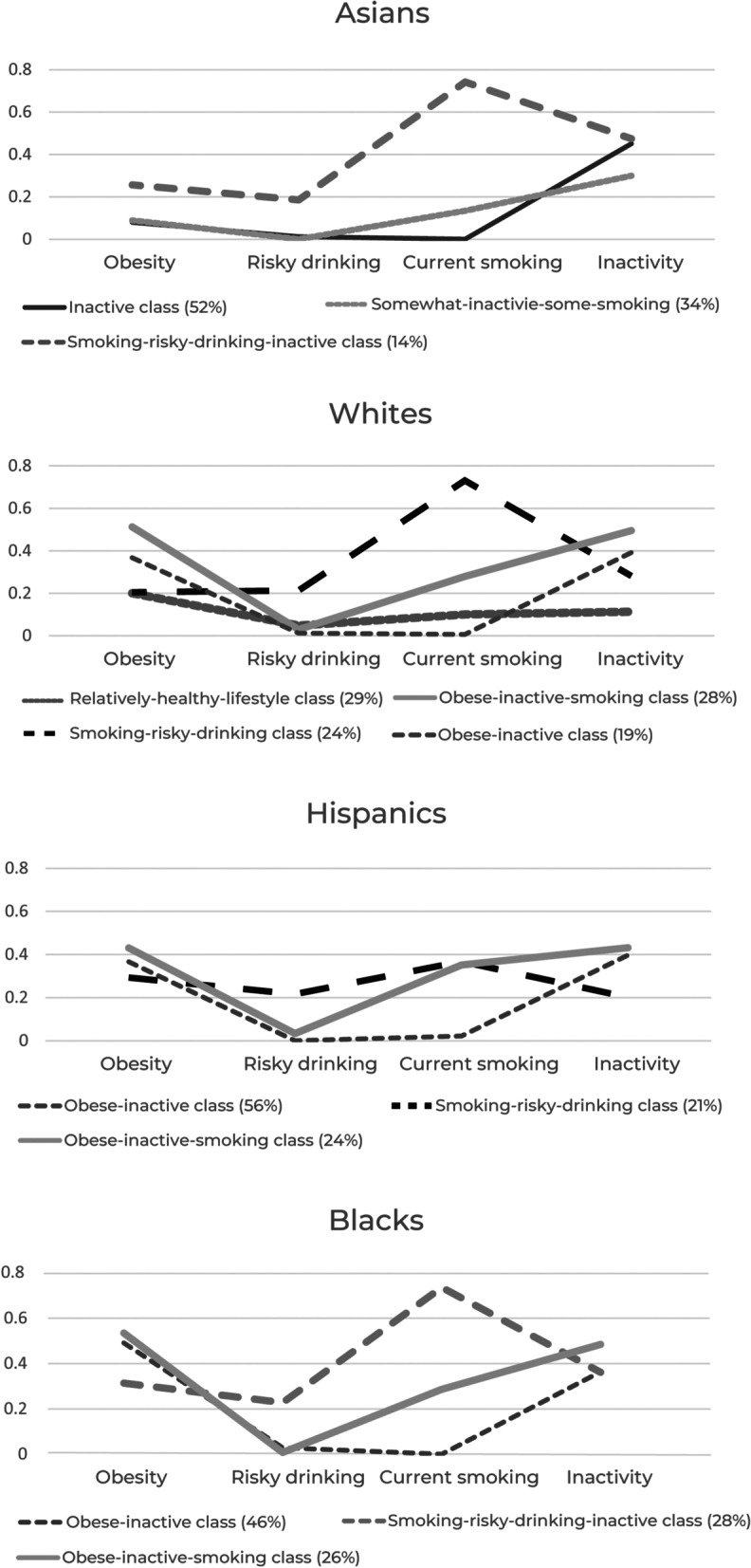
Classes of Clustered Risk Behaviors among US adults ages 40–70, NESARC Waves 2 and 3

As shown in Fig. 1, about three in ten White adults were in the *relatively-healthy-lifestyle class* characterized by low prevalence of risky drinking (4.8%), smoking (10.1%), and physical inactivity (11.3%), and relatively low prevalence of obesity (20.1%). The *obese-inactive class* (19%) had higher prevalence of obesity (36.8%) and inactivity (39.2%) but mostly did not engage in risky drinking (1.2%) or smoking (0.6%). The *obese-inactive-smoking class* (28%) had even higher prevalence of obesity (51.3%) and inactivity (49.6%), prevalence of current smoking (27.9%) somewhat higher than the overall average (25.7%; see Table 2), and very low prevalence of risky drinking (3.0%). The *smoking-risky-drinking class* (24%) had very high prevalence of smoking (73.1%) and relatively high prevalence of risky drinking (21.3%), and prevalence of obesity (20.5%) and inactivity (28.2%) somewhat lower than the overall and White averages.

**Table 2:** Latent class analyses of clustered risk behaviors: fit indices

Class	AIC	BIC	aBIC	Bootstrapped LRT
**Whites**				
2	145,965.496	146,100.501	146,046.475	*p* = .0000
3	145,479.29	145,685.76	145,603.141	*p* = .0000
4	145,233.189	145,511.14	145,399.912	*p* = .0000
5	145,191.133	145,540.557	145,400.727	*p* = .0000
**Blacks**				
2	54,688.53	54,806.07	54,752.047	*p* = .0000
3	54,406.844	54,586.611	54,503.988	*p* = .0000
4	54,374.325	54,616.318	54,505.095	*p* = .0000
**Hispanics**				
2	40,342.926	40,456.297	40,402.276	*p* = .0000
3	40,250.215	40,423.606	40,340.986	*p* = .0000
4	40,240.364	40,437.775	40,326.555	*p* = .0000
**Asians**				
2	7746.951	7834.752	7780.751	*p* = .0000
3	7727.185	7861.468	7778.879	*p* = .0000
4	7724.755	7905.52	7794.342	*p* = .0400

*AIC* Akaike information criterion, *BIC* Bayesian information criterion, *aBIC* Sample-sized adjusted BIC, *LRT* Likelihood ratio tests

For Black adults, almost half were in the *obese-inactive class* with about 49.1% of this class likely to be obese and 36.4% inactive, but hardly likely to smoke or engage in risky drinking. The *obese-inactive-smoking class* (25.0%) showed higher prevalence of obesity (58.5%) and inactivity (48.5%) than for the *obese-inactive class*, somewhat higher prevalence of smoking (28.6%) than the overall and group averages, and virtually no risky drinking (0.8%). The *smoking-risky-drinking-inactive class* (28%) had very high prevalence of smoking (73.6%) and relatively high prevalence of risky drinking (22.7%), and prevalence of inactivity (36.1%) somewhat higher than the overall average (32.3%).

For Hispanic adults, over half of them were in the *obese-inactive class* with about 36.6% of this class likely to be obese and 39.7% physically inactive, but not likely to smoke or engage in risky drinking. Like Whites and Blacks, the *obese-inactive-smoking class* (24.0%) had higher prevalence of obesity (43.1%) and inactivity (43.2%) than for the *obese-inactive* class, relatively high prevalence of smokers (35.2%), and very low prevalence of risky drinking (3.3%). The *smoking-risky-drinking class* was characterized by relatively high prevalence of smoking (36.8%) and risky drinking (21.6%), and lower than national and group averages of obesity (29.3%) and inactivity (20.2%).

About half of Asian adults were in the *inactive class*, with about 45.1% them likely to be inactive, but with low prevalence of risky drinking (1.2%) and obesity (8.0%), and no smokers. The *somewhat-inactive-some smoking class* (34%) had a similar profile to that of the *inactive class* but had some smokers (13.4%) and lower prevalence of inactivity (30.0%) than the *inactive class*. Lastly, the *smoking-risky-drinking-inactive class* had very high prevalence of smoking (74.2%), high prevalence of inactivity (47.4%), relatively high prevalence of risky drinking (18.5%), and prevalence of obesity (25.7%) lower than the overall average (33.7%).

### Associations of health conditions and SES with class membership: multinomial and logistic regressions

In multinomial logistic regression models to examine demographic and socioeconomic profiles of class membership (Table 3), the class deemed to show a healthier lifestyle than the other classes in each group was used as the referent. For Whites, all the other classes were likely to have lower income (aOR = 0.78, *p* < .001 for the *obese-inactive class*; and aOR = 0.75, *p* < .001 for the *obese-inactive-smoking class*) and education levels (aOR = 0.44, *p* < .001 for the *obese-inactive class*; and aOR = 0.14, *p* < .001 for the *obese-inactive-smoking class*) than the *relatively-healthy-lifestyle class*. For Blacks, the *smoking-risky-drinking-inactive class* had lower income (aOR = 0.85, *p* < .001) and education (aOR = 0.27, *p* < .001), and the *obese-inactive-smoking class* had lower education (aOR = 0.66, *p* < .01), than the *obese-inactive class*, the referent. For Hispanics, the *smoking-risky-drinking class* had lower education (aOR = 0.48, *p* < .05), but the *obese-inactive-smoking class* (aOR = 2.19, *p* < .001) and the *smoking-risky-drinking class* (aOR = 4.71, *p* < .001) had higher income, than the *obese-inactive class*. For Asians, the *smoking-risky-drinking-inactive class* had lower education (aOR = 0.06, *p* < .01) and income (aOR = 0.68, *p* < .001), but the *somewhat-inactive-some-smoking class* had higher income (aOR = 1.30, *p* < .001) than *the inactive class*. Compared to the *obese-inactive class* among Blacks and Hispanics (and the *inactive class* among Asians), other lifestyle classes were more likely to be male. Similarly, the *obese-inactive class* was less likely to be male for Whites. The *smoking-risky-drinking class* was younger compared to the referent among Whites and Hispanics.

**Table 3:** Demographic Profiles of Clustered Risk Behavior Classes among US adults ages 40–70

	Age	Male^a^	Married^b^	College degree + ^c^	Family income	US-born^d^
	aOR (95% CI)	aOR (95% CI)	aOR (95% CI)	aOR (95% CI)	aOR (95% CI)	aOR (95% CI)
**Whites** (*n* = 20,774)						
Relatively-healthy-lifestyle class (Ref)	–	–		–	–	–
Obese-inactive class	1.02 (1.00–1.04)*	0.42 (0.31–0.57)***	1.16 (0.86–1.55)	0.44 (0.35–0.57)***	0.78 (0.75–0.82)***	–
Obese-inactive-smoking class	1.08 (1.06–1.09)***	1.35 (1.06–1.09)*	0.90 (0.59–1.16)	0.14 (0.10–0.18)***	0.75 (0.71–0.79)***	–
Smoking-risky-drinking class	0.98 (0.97–0.998)*	2.29 (1.82–2.89)***	0.38 (0.30–0.50)***	0.10 (0.08–0.13)***	0.75 (0.71–0.79)***	–
**Blacks** (*n* = 7436)						
Obese-inactive class (Ref)	–	–	–	–	–	–
Obese-inactive-smoking class	1.08 (1.07–1.09)***	1.67 (1.34–2.08)***	0.83 (0.68–1.01)	0.66 (0.51–0.86)**	1.00 (0.96–1.04)	–
Smoking-risky-drinking-inactive class	1.00 (0.99–1.01)	4.05 (3.24–5.05)***	0.49 (0.39–0.62)***	0.27 (0.19–0.38)***	0.85 (0.79–0.92)***	–
**Hispanics** (*n* = 5819)						–
Obese-inactive class (Ref)	–	–	–	–	–	–
Obese-inactive-smoking class	1.04 (1.03–1.05)***	2.76 (2.21–3.45)***	0.76 (0.63–0.93)**	0.78 (0.55–1.10)	2.19 (1.69–2.83)***	0.97 (0.92–1.03)
Smoking-risky-drinking class	0.94 (0.91–0.96)***	10.27 (6.48–16.28)***	0.43 (0.30–0.64)***	0.48 (0.25–0.95)*	4.71 (3.19–6.94)***	1.03 (0.95–1.13)
**Asians** (*n* = 1293)						
Inactive class (Ref)	–	–	–	–	–	–
Somewhat-inactive-some smoking class	0.99 (0.95–1.04)	18.73 (8.72–40.24)***	0.86 (0.33–2.21)	1.89 (0.81–4.39)	1.30 (1.14–1.50)***	5.33 (1.13–25.24)*
Smoking-risky-drinking-inactive class	0.98 (0.93–1.05)	41.09 (13.71–123.65)***	0.32 (0.14–0.74)**	0.06 (0.01–0.45)**	0.69 (0.48–0.996)*	16.34 (5.09–52.52)***

*aOR* adjusted odds ratio, *CI* confidence interval

^a^Female as reference category

^b^Never married/separated/divorced/widowed as reference category

^c^No 4-year college degree as reference category

^d^Foreign-born as reference category

^***^p < 0.001

^**^p < 0.01

^*^p < .05

In logistic regression models to examine the associations between class membership and the two conditions (Table 4), the *obese-inactive class* was the referent for Whites as well, because of no individual with diabetes being in the *relatively-healthy-lifestyle class* and our intention to examine whether an additional risk behavior added to the most-commonly observed combination of obesity and inactivity was associated with higher odds of diabetes or hypertension. For Whites, the *obese-inactive-smoking class* was associated with both diabetes (aOR = 2.14, *p* < .001) and hypertension (aOR = 1.74, *p* < .001). The *relatively-healthy-lifestyle class* was associated with no odds for diabetes and lower odds for diabetes (aOR = 0.17, *p* < .001 for diabetes), and the *smoking-risky-drinking class* was also inversely associated with diabetes (aOR = 0.17, *p* < .001) and hypertension (aOR = 0.55, *p* < .001). For Blacks, the *obese-inactive-smoking class* was positively associated with diabetes (aOR = 1.43, *p* < .01) and hypertension (aOR = 1.40, *p* < .01), and the *smoking-risky-drinking-inactive class* was inversely associated with diabetes (aOR = 0.54, *p* < .001), compared to the *obese-inactive class*. For Asians, the *smoking-risky-drinking-inactive class* was more likely to be diabetic (aOR = 2.67, *p* < .05) than the *inactive class*. For Hispanics, there were no significant associations between class membership and either condition.

**Table 4:** Associations between Clustered Risk Behavior Classes and Chronic Conditions among US adults ages 40–70

	Whites (*n* = 20,774)	Blacks (*n* = 7436)	Hispanics (*n* = 5819)	Asians (*n* = 1293)
	aOR (95% CI)	aOR (95% CI)	aOR (95% CI)	aOR (95% CI)
**Diabetes**				
Obese-inactive class (Ref)	–	–	–	–
Relatively-healthy-lifestyle class	0.00 (0.00–0.00)***	–	–	–
Obese-inactive-smoking class	2.14 (1.64–2.79)***	–	–	–
Smoking-risky-drinking class	0.17 (0.09–0.32)***	–	–	–
Obese-inactive class (Ref)	–	–	–	–
Smoking-risky-drinking-inactive class	–	0.54 (0.41–0.71)***	–	–
Obese-inactive-smoking class	–	1.43 (1.10–1.86)**	–	–
Obese-inactive class (Ref)	–	–	–	–
Obese-inactive-smoking class	–	–	1.25 (0.93–1.67)	–
Smoking-risky-drinking class	–	–	0.68 (0.38–1.22)	–
Inactive class (Ref)	–	–	–	–
Somewhat-inactive-some smoking class	–	–	–	1.06 (0.32–3.44)
Smoking-risky-drinking-inactive class	–	–	–	2.67 (1.08–6.61)*
**Hypertension**				
Obese-inactive class (Ref)	–	–	–	–
Relatively-healthy-lifestyle class	0.27 (0.20–0.36)***	–	–	–
Obese-inactive-smoking class	1.74 (1.43–2.13)***	–	–	–
Smoking-risky-drinking class	0.55 (0.45–0.67)***	–	–	–
Obese-inactive class (Ref)	–	–	–	–
Smoking-risky-drinking-inactive class	–	0.96 (0.78–1.18)	–	–
Obese-inactive-smoking class	–	1.40 (1.13–1.75)**	–	–
Obese-inactive class (Ref)	–	–	–	–
Obese-inactive-smoking class	–	–	1.20 (0.94–1.53)	–
Smoking-risky-drinking class	–	–	0.85 (0.52–1.39)	–
Inactive class (Ref)	–	–	–	–
Somewhat-inactive-some smoking class	–	–	–	1.16 (0.54–2.49)
Smoking-risky-drinking-inactive class	–	–	–	0.83 (0.38–1.80)

Controlling for age, gender, income, education, US nativity, and health insurance coverage

*aOR* adjusted odds ratio, *CI* confidence interval

^***^p < 0.001

^**^p < 0.01

^*^p < .05

## Discussion

Our findings partially support our hypotheses: with some exceptions, we found unhealthier clustering of risk behaviors was associated with lower SES, and with chronic conditions. Common and different patterns were found in these relationships across the four racial/ethnic groups. Commonalities include the *obese-inactive class* among all but Asian adults (who, instead, had the *inactivity class*) and the clusters that add smoking to this mix in each group. Also common is the *smoking-risky-drinking class* among Whites and Hispanics, with a variation seen among Blacks and Asians in the addition of inactivity to this cluster. Key differences across racial/ethnic groups, or, to be precise, between Whites and racial minority groups, include: a sizeable *relatively-healthy-lifestyle class* observed only among Whites; and positive associations of unhealthier clusters with diabetes and hypertension, as well as with income and education, being more consistent for Whites than for others. For racial minority groups, education than income was more consistently associated with unhealthier clusters, and the associations of unhealthier clusters with the two disease conditions were less clear for Blacks and Asians than for Whites, with no significant association observed for Hispanics. As we discuss below, both the commonalities and differences across the groups have important public health implications.

The commonality of the *obese-inactive* cluster among Whites, Blacks, and Hispanics in the US suggests that addressing obesity and inactivity should be a key component of lifestyle interventions for these groups. Obesity is a well-recognized health problem in the US that increases risk for morbidity and premature mortality from major illnesses including hypertension, dyslipidemia, type 2 diabetes, cardiovascular diseases, respiratory problems, and some cancers [46]. As physical inactivity is one of the primary contributors to the obesity epidemic in the US [72], the common cluster of obesity and physical inactivity is not entirely surprising. However, another common cluster we found that additionally includes smoking is notable. As reported in a study, the joint effects of smoking, physical inactivity, and obesity could increase all-cause and CVD-specific mortality by at least 7.9 years U.S. adults [73]. The *obese-inactive-smoking class,* comprising about one in four adults in each of these three groups, is thus of great public health concerns.

The absence of a healthy lifestyle class among racial/ethnic minority groups does not mean that there were no individuals in these groups showing all four health-promoting behaviors, but the lack of such a class consisting of at least 5% of each minority sample in our LCA. This absence can be attributed to various sociocultural and structural forces. Past research suggests that the commonality of inactivity observed in all unhealthy clusters for Blacks and Asians may be partly attributed to lower social support for regular exercise in some Black communities [24] and Asian cultural values that place a lower priority on physical activity [27]. Perhaps more fundamentally, disparities in health-promoting and deleterious resources and environments (e.g., recreational spaces, food deserts, alcohol outlets) combined with differential exposure to chronic stressors such as racial discrimination [28] and financial strain [74], may lead to disparities in health behaviors [25, 26].

Overall, positive associations between unhealthier lifestyle classes and health conditions were more consistent for Whites than for others, with the *relatively-healthy-lifestyle class* having lower odds of diabetes and hypertension than the *obese-inactive class* (and the *smoking-risky-drinking class*, according to our post hoc analysis using the *relatively-healthy-lifestyle class* as the referent; results not shown for brevity of recording). Unhealthier clustering that adds smoking to the obesity-inactivity combination for Whites and Blacks (and to inactivity in Asians) was associated with higher odds of disease condition. To the extent these classes capture each respondent’s long-term lifestyle, these findings suggest elevated health risk associated with an additional risk behavior added to the unhealthy cluster. This is consistent with past research showing poorer health associated with larger counts of risk behaviors [75] or lower healthy lifestyle index scores [5, 6].

Still, given the cross-sectional design of the present study, strictly speaking, these associations capture continued engagement in risk behaviors while having either or both conditions, rather than the causal effects of the clustered risk behaviors on the conditions. That individuals who already have diabetes or hypertension and who can risk complications are *more*, *not less*, likely to (continue to) engage in risk behaviors is a cause for public health concerns. Concerted efforts to address clustered health risk behaviors in most US adults, particularly in those whose health conditions (such as diabetes and hypertension) are adversely affected by them, are warranted.

The *smoking-risky-drinking-inactive class* among Blacks and the *smoking-risky-drinking class* among Whites were associated with lower odds of disease conditions for Whites and Blacks, compared with the *obese-inactive class*. At least for Whites, this may be because of the lower age of the *smoking-risky drinking class* than the *obese-inactivity class* (Table 3)*,* given that these conditions tend to develop later in adulthood. More fine-grained analyses for different age groups among midlife or older adults may shed light on this, which we did not have sufficient statistical power to do.

We found unhealthier clustering mostly associated with lower SES. This pattern was the most consistent for Whites, with the other classes having lower income and education than the *relatively-healthy-lifestyle class*. This aligns well with prior studies that reported on positive associations between SES and healthier lifestyle using predominantly-White samples [2, 76]. For racial minority groups, education more than income tended to be associated with unhealthier clusters. For Blacks, for example, the *smoking-risky-drinking-inactive class* and the *obese-inactive-smoking class* had lower education (and the former had lower income as well) than the *obese-inactive class*. Similarly, the *smoking-risky-drinking-inactive class* among Asians and the *smoking-risky-drinking-inactive class* among Hispanics had lower education than their respective referents featuring fewer risk behaviors. Higher income, on the other hand, was positively associated with the clusters involving smoking and/or risky drinking among Asians and Hispanics, perhaps due to greater affordability of alcohol and tobacco that a higher income allows [77]. Each SES indicator measures different, often-related aspects of social stratification that may influence health [52]. Education influences health through a person’s adult occupation and income and the knowledge, health literacy and skills attained through education, which enable or motivate them to have healthier lifestyles [78]. It has been suggested that education gives individuals the ability to override the ‘default’ American lifestyle characterized by poor diet and inactivity [79]. Income can influence a wide range of material circumstances that affect health and access to health enhancing resources [78], but higher income alone may have limited salutogenic effects, particularly for racial minority groups.

That the relationship between higher SES and healthier lifestyle is more pronounced for Whites than other groups may be partly because cultural practices and social support (or the lack thereof for healthy lifestyle) in racial minority groups, which, as we noted above, may also influence lifestyle independently of SES to some degree. Unhealthy lifestyles among racial minority groups may also be attributed to psychosocial stressors such as racial inequities they experience (including low occupational achievements even at the same education level as Whites) [80], racial prejudice and unfair treatment they encounter in everyday lives [28], and overall lower subjective social status they may experience [81, 82], all of which may trigger health risk behaviors to cope with these stressors [74] or distract individuals from health-seeking behaviors [25, 26].

Our findings have important implications for future interventions. Given that the *obese-inactive class* and the *obesity-inactive-smoking class* among White, Black, and Hispanic adults together comprise a large segment of each group—47% of White, 72% of Black, and 80% of Hispanic adults (Fig. 1)—obesity and inactivity should be a central focus of lifestyle interventions for these three groups. Furthermore, in light of the synergistically adverse health effect of obesity, inactivity, and smoking on health noted above [73], as well as our current findings showing positive associations of the *obese-inactive-smoking class* with diabetes and hypertension for Whites and Blacks, preventive and intervention strategies for maintaining good cardio-respiratory health, particularly for these two groups, are warranted. Importantly, it should be emphasized that while clinical patient-oriented interventions are important, multi-level interventions are very likely needed for facilitating behavioral change, as health risk behaviors are related to individual, neighborhood, and environmental conditions as noted above.

In light of the consistently inverse associations between SES and unhealthy lifestyles for White adults, targeting low-SES Whites with these intervention strategies might be fruitful. Interventions to address obesity and inactivity might be directed most adults among Blacks and Hispanics, regardless of their SES. Still, given the significant negative association of the *obese-inactive-smoking class* and college degree for Blacks, it would be reasonable to target Blacks without a college degree with interventions addressing all three behaviors. The absence of obesity in any unhealthy lifestyle cluster and the commonality of inactivity in unhealthy clusters for Asians calls for a unique strategy to improve their health behaviors. As noted above, physical inactivity may be rooted in Asian cultural values that do not emphasize exercise, and thus interventions to address them could be effective, especially when considering the additional health risks of smoking alongside inactivity. While inactivity is also present in all unhealthy lifestyle classes among Blacks, the high prevalence of obesity among Blacks warrant strategies to address both obesity and inactivity.

We acknowledge several limitations of this study. First, gender differences in the clustering of risk behaviors were not explored in our LCAs to maximize statistical power for racial/ethnic-specific analyses and also to keep the focus on racial/ethnic differences. Still, multinomial regressions captured gendered clustering of risk behaviors (e.g., the *obese-inactive class* more likely to be female). Second, though reasonable [44], the use of obesity as a proxy for unhealthy diet is a limitation. Third, as our LCA models were not specific to gender, we used the cutoff for risky drinking for men (> 14 drinks weekly versus > 7 drinks weekly for women) largely because risky drinking is more pervasive among men than women [83]. Using a conservative measure for women’s heavy drinking may have underestimated the unhealthy clusters involving risky drinking among them. There may indeed be gender differences in the clustering of risk behaviors, which we did not explore in order to maximize statistical power for racial/ethnic-specific analyses and also to keep the focus on racial/ethnic differences.

## Conclusions

The current study meaningfully contributes to the health disparities literature concerning health risk behaviors. Adding to prior studies that used quantitative summary measure of the clustering of risk behaviors [4–6], our study provides qualitative information on the actual clustering and for each racial/ethnic group. Our race-specific findings—e.g., the absence of a (relatively) healthy lifestyle class and more consistent associations of unhealthier clustering with education than with income among racial minorities—add important nuances to the thesis of higher SES accompanied by a healthier lifestyle, established in studies using predominantly White samples [2, 76]. In doing so, this study points to the need to better understand the complex pathways by which social determinants influence health in ways that are common and diverse across racial/ethnic groups. Additionally, the common and diverging patterns of clustering across these groups and their associations with the two chronic conditions we report have the great potential to inform multi-behavior interventions. Concerted efforts to address clustered risk behaviors in most US adults, particularly racial/ethnic minority groups and those with chronic conditions, are warranted.

## Data Availability

Not applicable. This study used national survey data NIAAA makes available upon request.

## Abbreviations

AIC: Akaike information criterion
BIC: Bayesian information criterion
LCA: Latent class analysis
NESARC: National Epidemiologic Survey on Alcohol and Related Conditions
SES: Socioeconomic status
US: United States
